# Challenges and Opportunities for Mental Health and Psychosocial Support in the COVID-19 Response in Africa: A Mixed-Methods Study

**DOI:** 10.3390/ijerph19159313

**Published:** 2022-07-29

**Authors:** Alice Walker, Muhammad Abdullatif Alkasaby, Florence Baingana, William K. Bosu, Mohammed Abdulaziz, Rosie Westerveld, Adelard Kakunze, Rosemary Mwaisaka, Khalid Saeed, Namoudou Keita, Ian F. Walker, Julian Eaton

**Affiliations:** 1 UK Public Health Rapid Support Team, UK Health Security Agency/London School of Hygiene & Tropical Medicine, London WC1E 7HT, UK; alice.lj.walker@googlemail.com (A.W.); ian.walker1@dhsc.gov.uk (I.F.W.); julian.eaton@lshtm.ac.uk (J.E.); 2 Centre for Global Mental Health, London School of Hygiene and Tropical Medicine, London WC1E 7HT, UK; 3 Department of Infectious Disease Epidemiology, Faculty of Epidemiology and Population Health, London School of Hygiene & Tropical Medicine, London WC1E 7HT, UK; 4 WHO Regional Office for Africa, Brazzaville P.O. Box 06, Democratic Republic of the Congo; bainganaf@who.int; 5 West African Health Organisation, Bobo-Dioulasso 01 BP 153, Burkina Faso; wbosu@wahooas.org (W.K.B.); nkeita@wahooas.org (N.K.); 6 Africa Centres for Disease Control and Prevention, African Union, Addis Ababa P.O. Box 3243, Ethiopia; mohammedab@africa-union.org (M.A.); kakunzea@africa-union.org (A.K.); 7 Independent Researcher, 24620 Les Eyzies de Tayac, France; rosiewesterveld@gmail.com; 8 East, Central and Southern Africa Health Community, Arusha P.O Box 1009, Tanzania; rmwaisaka@ecsahc.org; 9 WHO Regional Office for the Eastern Mediterranean, Cairo 11371, Egypt; saeedk@who.int; 10 Office for Health Improvement and Disparities, Department of Health and Social Care, London SW1H 0EU, UK; 11 CBM Global Disability and Inclusion, 1181 LE Amstelveen, The Netherlands

**Keywords:** mental health and psychosocial support, emergency preparedness and response, COVID-19, infectious disease outbreaks, Africa

## Abstract

This research aimed to (1) assess the extent to which mental health and psycho-social support (MHPSS) was included in the national response to the COVID-19 pandemic in African countries, and (2) explore barriers and enablers to MHPSS integration into the COVID-19 response. A mixed-methods study, using an online survey and in-depth interviews, was conducted. Participants included Mental Health Focal Points at the Ministries of Health, the World Health Organization (WHO) country and regional offices, and civil society representatives. Responses were received from 28 countries out of 55 contacted. The implementation level, based on standard guidelines, of MHPSS activities was below 50% in most countries. The most implemented MHPSS activities were establishing coordination groups (57%) and developing MHPSS strategy (45%), while the least implemented activities included implementing the developed MHPSS strategy (32%) and establishing monitoring and evaluation mechanisms (21%). Key factors that hindered implementing MHPSS activities included lack of political commitment and low prioritisation of mental health during emergencies, as it was seen as a “less important” issue during the COVID-19 pandemic, when more importance was given to infection prevention and control (IPC). However, there are signs of optimism, as mental health gained some attention during COVID-19. It is imperative to build on the attention gained by integrating MHPSS in emergency preparedness and response and strengthening mental health systems in the longer term.

## 1. Introduction

The COVID-19 pandemic has had a massive impact on people’s mental health worldwide [1], with evidence that the prevalence of major depressive and anxiety disorders increased by a quarter in 2020 alongside high rates of reported distress [2,3]. In a global survey of 130 countries conducted by the World Health Organization (WHO), 93% of the countries reported substantial disruption in their services for mental, neurological, and substance use disorders during the pandemic [4]. This is of particular concern given that mental health services in many countries were already fragile and poorly funded [5]. Although 89% of countries that responded to the previous WHO survey reported that MHPSS was part of their national COVID-19 response plans, only 17% had ensured that full additional funding is available for MHPSS activities [4]. This suggests a substantial gap exists between the planning for and implementation of MHPSS activities. Additionally, the extent to which those plans are implemented was not clear.

International normative guidance on MHPSS in the COVID-19 pandemic was developed quickly in the early months of the pandemic, in 2020. For instance, the Inter-Agency Standing Committee (IASC) Reference Group on MHPSS in Emergency Settings issued an interim briefing note to outline the MHPSS aspects of the COVID-19 outbreak [6]. This guidance outlined fourteen key activities that were recommended to be implemented as a part of the national COVID-19 response.

This study aimed to (1) assess the extent to which the IASC recommended MHPSS activities were included in the national response to the COVID-19 pandemic in African countries and (2) explore barriers and enablers to MHPSS integration into the COVID-19 response. Identifying challenges and opportunities for MHPSS integration into the COVID-19 response will provide evidence to inform preparedness and response to future infectious disease outbreaks and other public health emergencies in the continent.

## 2. Methods

### 2.1. Study Design and Participants

An explanatory sequential mixed-methods study, using an online survey followed by in-depth interviews, was conducted with data collection taking place between October and December 2020. In this approach, initial quantitative results are followed up by more in-depth qualitative research to better understand quantitative results.

In order to obtain data on MHPSS response in African countries, we purposively selected Mental Health Focal Points in national Ministries of Health (MoH), WHO Regional and Country Offices, and staff of National Public Health Institutions to respond to the survey and to be interviewed. Civil society representatives were also included in the interviews to obtain information from different perspectives. The invitation to participate was sent via Africa Centres for Disease Control and Prevention (Africa CDC), West African Health Organisation (WAHO) and WHO Regional Office for Africa (AFRO), and WHO Regional Office for the Eastern Mediterranean (EMRO)—all of whom are members of the research consortium in the “Strengthening Public Mental Health in Africa in response to the COVID-19 Epidemic (SPACE)” project.

### 2.2. Data Collection

A web-based questionnaire was developed to assess the participants’ perception of the degree of implementation of the IASC MHPSS Reference Group’s ‘14 Globally Recommended Activities’ [6] according to a 4-point Likert scale (fully implemented, almost fully implemented, somewhat implemented, and not implemented at all). The survey also included open-ended questions about participants’ experience of efforts to progress the MHPSS agenda as part of the COVID-19 response. (Box 1) provides the thematic areas and questions of the survey. The full questionnaire (Supplementary File S1) was translated into French and Portuguese to facilitate equitable participation and maximise response rate, then sent to WHO and the Ministry of Health in each African Union member state [7]. After that, reminder emails were used to boost responses from under-represented regions to ensure a minimum of 25% response rate across all regions.

Box 1 **Survey thematic areas and questions based on IASC’s recommended actions.**

1. **Assessing Need**

Q1: To what extent has a rapid MHPSS needs assessment been performed as part of the COVID-19 response in your country?

2. **Coordinating Action**

Q2: To what extent has an MHPSS coordination group, technical working group, or response unit been established?

Q3: To what extent has an MHPSS strategy for COVID-19 been developed?

Q3b: To what extent has this strategy been implemented?

Q4: To what extent has information from needs assessments and service analysis been used to ensure there is a system to identify and provide care to people with common and severe mental health and substance misuse conditions?

3. **Delivering Support**

Q5: To what extent have MHPSS activities been integrated into other sectoral response activities?

Q6: To what extent are there functional referral pathways to MHPSS services from other sectors

Q7: To what extent are accurate information and communication materials on MHPSS available and accessible?

Q8: To what extent have frontline workers been trained in the principles of psychosocial care and psychological first aid?

Q9: To what extent do frontline workers responding to COVID-19 have access to psychological support?

Q10: To what extent has specific support for children and families been established as part of the COVID-19 response?

Q11: To what extent have community-based forms of MHPSS been established?

Q12: To what extent have MHPSS measures been established to reduce the negative impact of social isolation?

Q13: To what extent have COVID-19 and MHPSS protocols been developed for funerals and mourning?

4. **Monitoring and Evaluation**

Q14: To what extent have monitoring and evaluation mechanisms to measure MHPSS activities been established?

To explore barriers and enablers to integrating MHPSS activities into the COVID-19 response, we conducted in-depth semi-structured interviews, with 1–2 individuals in 12 selected countries, identified from the survey respondents. Those key informants were selected, in order to capture (a) countries which have different levels of experience in emergency response, (b) countries with different levels of human and financial resources, and (c) regional representativeness. We also used snowball sampling to identify other key stakeholders, including civil society actors involved in mental health. Although the aim was not formally to compare different needs and resources, we deliberately chose a variety of countries to draw learnings across different contexts in relation to resource levels and preparedness for emergency response. We drew from the established literature as well as the major barriers and facilitators identified from the survey to inform and shape the Interview Guide for the semi-structured interviews (Supplementary File S2). The semi-structured interviews were conducted by an experienced bilingual researcher, and recorded through the Zoom platform, with each interview lasting around 50 min. Questions focused on contextual factors, enablers and challenges in integrating MHPSS into the COVID-19 response. Participants were also asked specifically about enablers and barriers identified through the survey data. Ten interviews were conducted in English and seven in French. Interviews were translated and transcribed by an external contractor.

### 2.3. Data Analysis

#### 2.3.1. Quantitative Analysis

The survey data were downloaded into a Microsoft Excel spreadsheet from the web-based platform. Survey responses were subjected to descriptive analysis by region, language, and responding institution. The respondents’ perception of implementation strength was colour-coded to provide a visual summary of the results by country. In order to compare responses across countries and regions, responses were assigned a numerical score (1–4) based on a Likert scale response where one equals “not implemented at all” and four equals “Fully implemented”. “Do not know” and blank responses were assigned zero. Scores from all questions were added together, and this score was then converted into an “implementation percentage” (0–100%) to provide an overall numerical measure of implementation across all 14 Globally Recommended Activities. A similar technique was used to give an indication of the implementation score according to the specific IASC recommendation.

#### 2.3.2. Qualitative Analysis

Open-text responses from the survey were subjected to content analysis to derive a list of the major barriers and facilitators mentioned by the participants and were used to generate the topic guide for the in-depth interviews. Inductive thematic analysis was carried out using the NVivo qualitative analysis software [8] to allow for empirical findings to emerge. Thematic coding was developed, reflecting key themes identified as part of the research development and data collection and analysed in light of the research aims and questions. Coding focused on four key types of data:

- Thematic, including issues relating to programming and coordination, public health system, capacity in public mental health, resources, and existing guidance;
- Descriptive, notably around contextual information and stakeholder relationships;
- Informative, focusing on insights from the informants’ experiences, such as lessons learned, suggestions and recommendations, and best practices;
- Assessment, dealing with evaluative data such as positive, opportunity, enabler, facilitators; negative, challenge, constraint, gap, barriers; low/high priority.

### 2.4. Ethical Considerations

We obtained ethical approval from the London School of Hygiene & Tropical Medicine (LSHTM) Research Ethics Committee. The entire study was developed and implemented as part of a wide consortium with key African public health institutions and results were shared with participants as part of a programme to strengthen MHPSS preparedness and response on the continent. Written informed consent was obtained from all participants. Prior to participation, we shared information about the study and its aim with the participants. We explained that participation is voluntary, and they are free to withdraw at any time. Participants provided contact details for the purposes of follow-up and continued engagement in the capacity-building element of the research programme, but all responses were anonymised prior to data storage and analysis.

## 3. Results

### 3.1. Quantitative Results

**Figure 1:**
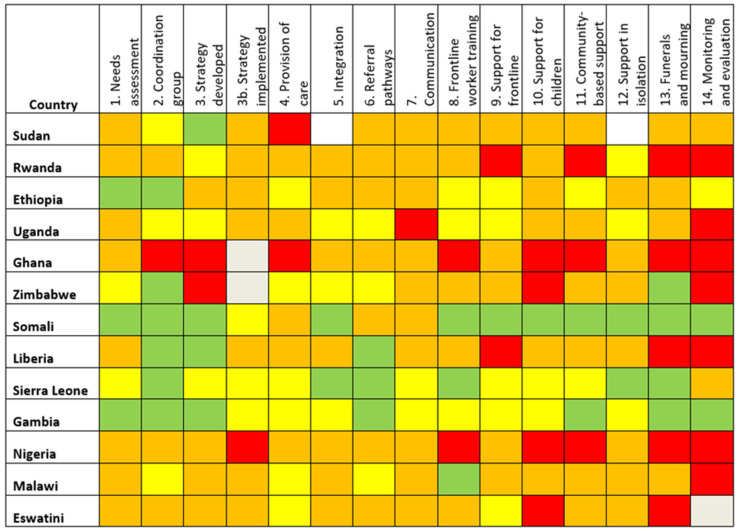
Anglophone countries’ responses to survey based on IASC 14 recommended MHPSS activities in the COVID-19 outbreak.

**Figure 2:**
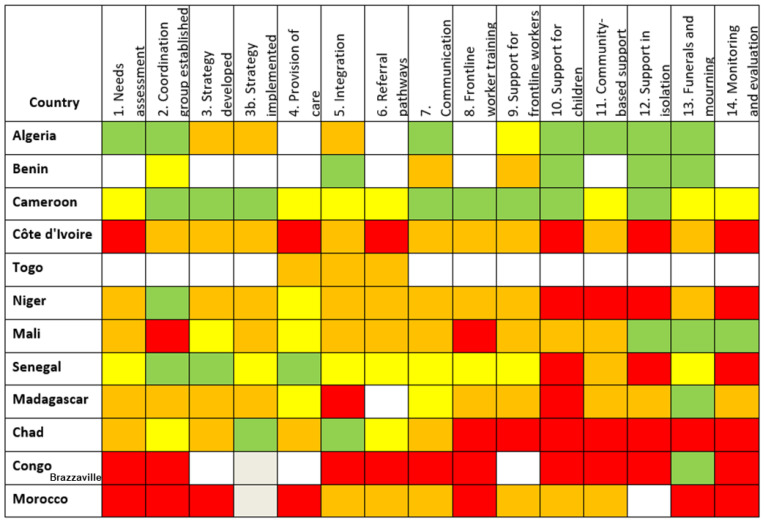
Francophone countries’ responses to survey based on IASC 14 recommended MHPSS activities in the COVID-19 outbreak.

**Figure 3:**
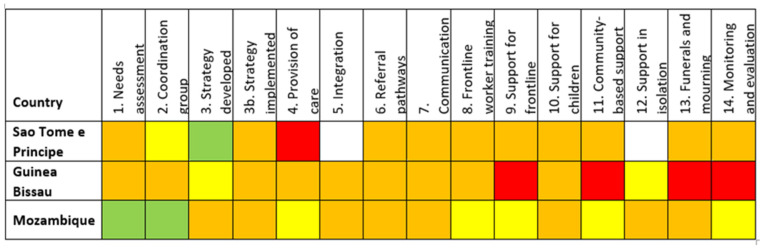
Lusophone countries’ responses to survey based on IASC 14 recommended MHPSS activities in the COVID-19 outbreak.

Responses were received from 28 out of 55 countries (51%) (Figure 1, Figure 2 and Figure 3). Thirteen (46%) of responding countries were lower-middle-income, and 54% were low-income. Thirteen (46%) of the responses were received in French (from countries where French was one of the official languages), 43% in English, and 11% in Portuguese. Response rate by region varied between 44% (North Africa region) and 86% (West Africa region). Regarding respondents’ workplace, 75% were affiliated with Ministries of Health, 11% were WHO mental health focal points, and 14% worked in National Public Health Institutes (which are themselves affiliated to Ministries of Health).

The colour-coded responses on the levels of implementation (Table 1) allowed the construction of visual dashboards organised by three linguistic blocks (Figure 1, Figure 2 and Figure 3).

**Figure 4:**
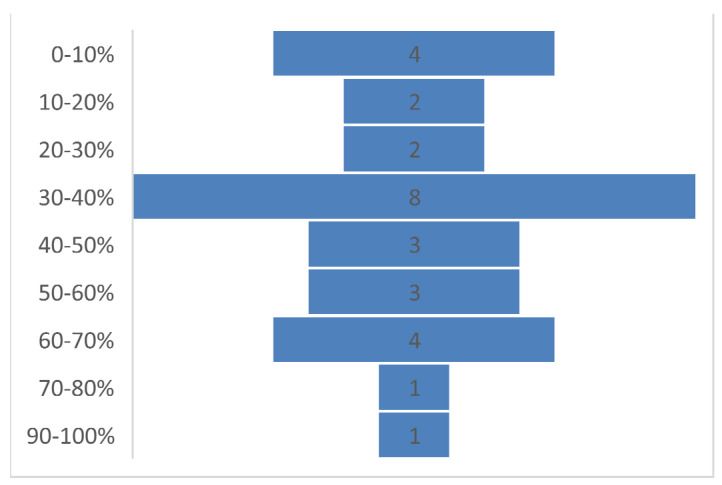
The number of countries at each implementation level.

The implementation level in most of the countries (19/28) was below 50% (Figure 4). There were no significant differences in implementation scores by region, income level, or responding institution. However, there were differences between the scores of anglophone, francophone, and lusophone respondents, with average scores of 44%, 38% and 22%, respectively. This was not tested for significance due to the small number of lusophone respondents.

**Figure 5:**
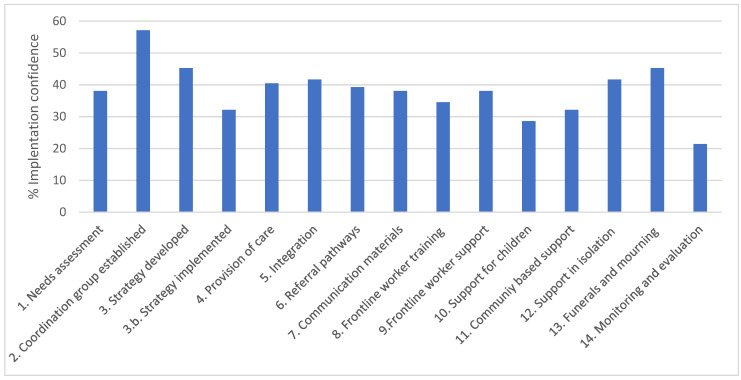
Implementation score by each IASC recommendation.

Recommendations implemented to the greatest degree were “Establishment of Coordination Group” (57% overall score), “Strategy Developed” (45%) and “Protocols for funerals and mourning developed” (45%). The lowest were “establishing monitoring and evaluation mechanisms” (21%), “Support for children” (28%), and “Community-based support” and “Strategy implemented” (both 32%) (Figure 5).

### 3.2. Qualitative Results

Seventeen in-depth interviews were carried out with participants from 12 countries. The evidence generated through the qualitative data collection and analysis highlighted some key challenges and opportunities for mental health in the context of emergencies, which we outline below.

#### 3.2.1. Challenges to Improving MHPSS Components in Crisis Response

##### Lack of Political Commitment and Engagement

Almost all participants felt there was a lack of engagement and interest in mental health—both before and at the outset of the COVID-19 pandemic. Participants described this as occurring at all levels; in their interactions with political leadership, health service management and community members. This lack of engagement subsequently plays a key role in the way that mental health is integrated—or not—within health, social and psychosocial interventions and funding, particularly in emergency contexts where decisions are being made rapidly. At a national level, participants described a lack of leadership and unwillingness of high-level personnel within ministries to prioritise mental health aspects of crisis response management. This led to a situation in which the responsibility for pushing the MHPSS agenda fell to a small number of staff, risking work overload and burnout.

*“We’venot got so many people who would want to take lead in managing mental health programmes at different levels. (…) When my colleagues were in quarantine, we looked for someone to take over as we rest maybe for a week or two and we could not find anyone”*.

[MoH Mental Health (MH) focal point]

Carrying out advocacy and lobbying influential leaders were described as some of the biggest challenges, absorbing most of the energy from high-level mental health staff.

##### Low Prioritisation of Mental Health within Emergency Response Structures

Respondents noted that Infection Prevention and Control (IPC) and Case Management get most of the attention during outbreaks. Mental health was consistently described as less of a priority than other issues seen as more pressing or urgent.

*“They prioritisecase management, infection prevention …, making sure everything else was ready but mental health was not given the priority with that, thus no fund was allocated to it”*.

[MoH MH focal point]

During the COVID-19 pandemic, the focus was initially on the physical manifestations of the disease, medical needs and advancements, morbidity and mortality, the economic impact of lockdown and the implementation of social distancing and protective measures.

##### Lack of Institutional Memory and Failure to Apply Lessons Learned

Despite lessons learned during previous outbreaks with high reported mental health impacts, such as Ebola Virus Disease, MHPSS has not been systematically prioritised in recent crisis responses. Respondents particularly noted this to be the case in preliminary planning, coordination and preparation of COVID-19 responses.

*“We havenot been able to take advantage of the past. There is still difficulty in giving the right place to mental health in the response to the COVID-19”*.

[Civil society representative]

Interviewees reported that existing infrastructures and services that had been developed to handle outbreaks were not maintained, which meant that they could not be used when new outbreaks occurred.

*“We usedsome of the systems and structures that were put in place for Ebola, but we clearly ignored some of them or flagrantly neglected to uphold some of them”*.

[Civil society representative]

Similarly, personnel who had gained skills and experience in previous emergencies were not identified and deployed in the pandemic (see below).

##### Lack of Funding

Funding deficits were consistently reported as a central challenge for MHPSS Leads in the set-up of their strategies, as well as service provision and response. Even when funding becomes available in some situations, participants expressed how the lack of financial sustainability from national and global partners has impeded the strengthening of mental health services in their settings in the post-emergency recovery phase.

*“These partnerscome on board when there’s an immense emergency, there is a pandemic, when there is a disaster. After that...they all leave and go away on different programmes and we struggle on our own”*.

[MoH MH focal point]

Without a budget explicitly dedicated to MHPSS, interviewees anticipate funding delays or changing agendas will impede crisis preparedness, as well as response, due to the impact on the availability of training, staff, medication, and reporting systems. Inadequate funding also led to support services (such as hotlines) not being operational or being overburdened.

When funding was made available by external stakeholders for the COVID-19 response, participants lamented that there was no specific budget line allocated specifically to mental health, leading to the deprioritisation of mental health activities:

*“Even thefunding that we have with WHO never came as funding for mental health in COVID. It comes as just the COVID response in particular targeting case management, IPC and the like”*.

[WHO MH focal point]

Lack of donor preparedness for emergencies impacted how funding was made available, which was not deemed sufficiently flexible for outbreak response. The timelines and protocols to reallocate budgets to pandemic relief efforts contributed to the spread of the virus, as reported by one of the participants:

*“Donors’ contributionsform a significant proportion of our overall budget and [when COVID-19 hit] no donor at the time had COVID activities planned. They all had different healthcare issues or projects that they were meant to support. But then COVID came as an unexpected thing. It wasn’t easy to have people repurpose money in the very beginning. And while we were going through all the bureaucracies to have that done, the virus was spreading in the communities and the government was still begging people here and there”*.

[Civil society representative]

##### Information Systems and Reporting

Planning and coordinating MHPSS response activities require updated data and robust information systems to monitor needs and evaluate response. Interviewees mentioned that issues with inadequate data collection and reporting processes hindered the understanding of the ongoing crisis in the field, thus impeding appropriate and timely response.

*“We didn’thave enough mental health indicators collected or people trained on how to fill this indicator, so we had inadequate data collection and reporting, so what you are getting—the reports we are getting [do not represent] a true picture of what’s going on in the ground”*.

[MoH MH focal point]

Interviewees also reported gaps in staff training on how to collect, process, and report available data. Participants associated the gaps experienced in data availability and collection on mental health to the weakness of health systems and the fact that mental health was not approached as an individual outbreak response pillar.

Moreover, During the COVID-19 pandemic, data collection efforts were impeded by high levels of stigma associated with being infected with the disease, as well as a rise in anxiety and fear about the possible effects of contamination. This led to a reluctance of community members to provide personal data.

Given the heterogeneity of actors involved in mental health, participants noted the need for integrated and collaborative approaches to mental health data collection and processing to increase understanding of mental health needs to enable better responses.

##### Human Resources Challenges

Participants across all contexts reported a severe lack of qualified staff in mental health or psychosocial support. When trained or qualified staff do exist in emergency scenarios, they may be deployed to other work deemed a higher priority.

*“The humanresources (…) who were trained on mental health were doing other things, like you’d find the [district] focal person for mental health is working in the antenatal clinic or is working in paediatrics, and is not there offering mental health support because they’re doing routine work”*.

[MoH MH focal point]

More broadly, mental health staff being neglected within the existing health schemes and payrolls was pointed out as a recurrent obstacle. According to participants, this explains why mental health personnel are not consistently spread out throughout the different regional administrations of a country—and why mental health professions are not considered attractive careers. A recurrent complaint was that staff are not being paid, are underpaid or are not paid consistently. When budget reforms occur, participants noted that mental health professions were commonly the first to lose funding.

*“Mentalhealth clinicians [are paid] little money and sometimes not paid at all, as compared to nurses and other people that were working in the same area. It was very noticeable, and it was demotivating for our colleagues in mental health…”*.

[Civil society representative]

Often, mental health professionals have to carry out their work either as volunteers or under other professional roles (for example, psychologists hired as mental health nurses).

*“In our teampsychologists are not integrated in the health system…they are volunteer, they have left their job in order to help the population…It is very important for us to have means to support them so that they will continue it”*.

[MoH MH focal point]

Training was commonly described as either missing or inadequate. One of the main concerns expressed by participants is that the existing training for healthcare workers is developed based on a medical curriculum that does not include mental health considerations such as psychosocial skills, public health issues or holistic care.

*“The trainingof the primary healthcare workers is also very medicalised. They are not trained in basic kinds of psychological, psychosocial skills to make the person feel comfortable, you know, get the most out of the person as possible”*.

[Civil society representative]

In the context of the COVID-19 pandemic, participants stressed the importance of training frontline responders in MHPSS techniques, as opposed to confining MHPSS expertise to mental health professionals.

When frontline personnel had been trained, participants reported that these staff were not included systematically or sustainably in emergency response in later emergencies, despite their qualifications and experience in handling outbreak responses. This led to the under-utilisation of skills and expertise, which in turn impacted funding allocation and response timelines as new staff needed to be trained and dispatched to the affected areas.

*“In someareas, we actually train healthcare workers in the delivery of psychosocial support. But what happened [during an Ebola outbreak] was that we did not go back to the same people—like community health volunteers and other healthcare workers—who had prior knowledge and experience in handling Ebola in the area to come on board right away”*.

[Civil society representative]

##### Communication Challenges

Internet communication infrastructures, the availability of technical devices such as phones or computers, and financial means to purchase internet data credit, impacted service coordination and accessibility. This was particularly important in the context of services provided remotely.

*“Thereare areas where their own network is extremely poor, the people cannot call even if the number is free of charge, it’s difficult for them to call because they don’t have network”*.

[MoH MH focal point]

Several participants also described the impact of poor telecommunications on response planning and coordination.

*“We weremeeting on Zoom in most cases, so if you do not have internet access, you will not be able to connect. It’s bad for us as a government entity, you would not be able to connect. And the partners are looking up to you as the government to call for a meeting, to arrange the meeting”*.

[MoH MH focal point]

##### Competing Priorities in Emergency Situations

Participants in some contexts highlighted the fact that competing priorities experienced by communities led to a sense of disengagement with efforts to control the disease. Despite initial fear and anxiety triggered by COVID-19, the pandemic was not considered a priority for some populations.

*“Thepopulation at one point in time no longer took the COVID-19 epidemic as a priority. When you compare it to other African countries or to Europe, people will say that they have many more problems than the COVID-19 epidemic. It wasn’t the priority, the population had other more pressing needs”*.

[Civil society representative]

With an initial focus on controlling and preventing the spread of the pandemic, governments concentrated on enforcing new protective and sanitary measures and did not anticipate the emotional and psychological effects of these measures.

*“First of all, psychologically, people are suffering... It’s very important to help them. Because we are talking about washing hands, distance, but the suffering of people, they don’t address it, how to manage it”*.

[MoH MH focal point]

The effects of quarantine and social distancing measures, including increased anxiety, fear, aggression, stress, insomnia and panic, were reported as main drivers of the need for MHPSS integration in the pandemic response. However, MHPSS activities were often only in quarantine settings, rather than being organised as effectively for the broader population affected by similar stressors.

Box 2 summarises the key challenges to integrating MHPSS into emergency response.

Box 2 **Challenges to integrating MHPSS into emergency response.**

- The lack of political commitment and engagement
- Low prioritisation of mental health within emergency response structures
- The lack of available and sustainable funding
- The lack of monitoring, evaluation, and reporting mechanisms.
- Failure to apply lessons learned from previous emergencies.
- Human resources challenges (e.g., shortage in trained MHPSS staff, underpaid staff)
- Communication challenges (e.g., poor telecommunication infrastructure)
- Competing priorities in emergency situations.

#### 3.2.2. Enablers and Opportunities in Improving MHPSS Components in Crisis Response

##### Capitalising on an Increased Political Will

Participants noted that the current pandemic has increased the attention on mental health issues, probably more than has ever been the case in previous outbreaks. The rise in the reported mental health issues among those affected by the pandemic facilitated public discussions on the emotional aspects of the outbreak by a broad range of stakeholders.

*“There werea lot of interventions in the media, involving political figures, religious leaders, doctors, nurses, at all levels to explain the disease to the population. It was really a surprise”*.

[WHO MH focal point]

The increased attention on mental health was seen as an opportunity to drive discussions on the importance of mental health inclusion in outbreak responses and address structural barriers such as funding or political engagement, which previously hindered integrated approaches.

*“As the pandemicspread and people started exhibiting anxieties, depressions and fear, that had a toll on their productivity and function, their thinking changed and the approach changed for the better. So I believe that in planning for future pandemics or emergencies, we would not lose sight of the key component mental health has to play in such situations”*.

[WHO MH focal point]

In some cases, previously halted or slow-progressing strategies such as mental health action plans or suicide prevention strategies received high-level attention and were deemed national priorities during the COVID-19 crisis.

*“COVID-19 has helped us fast track is we are working actually, we’re finalising ournational suicide prevention strategy and programme, and that’s top of the agenda for the Minister of Health because there were reports of suicidal attempts at the quarantine sites”*.

[MoH focal point]

##### Promoting MHPSS Integration in Emergency Response

Participants highlighted how previous outbreak responses were problematic when they failed to include MHPSS as an essential pillar with allocated budgets. The high rates of mental health issues during COVID-19 necessitated rapid interventions from mental health personnel and made policymakers realise the importance of including MHPSS into emergency preparedness and response plans.

*“Before, we didn’t have a section on MHPSS and emergencies in humanitarian setting, but nowwe’ve put that in our action plan so that we can plan, we can prepare for other disaster, not just COVID-19, and we can use what we’ve learned from COVID-19 response”*.

[MoH MH focal point]

##### Better Public Understanding of Mental Health following the Pandemic

The increased burden of COVID-19 on the population’s mental health encouraged public debate and discussions on mental health. Participants reported that COVID-19 had increased the interest in mental health at the community level and enabled different stakeholders to discuss mental health openly, allowing greater openness to addressing stigma and beliefs associated with mental health issues.

*“Peopleare really yearning for information [about mental health]… they really have been feeling uncertain. Then some are getting depressed and so on. So, we’re seeing people now appreciating that it’s possible for someone to feel that way no matter the results, so there’s a clear understanding of mental issues in communities”*.

[MoH MH focal point]

##### Integration of Mental Health in Routine Services and Strengthening Mental Health Systems in the Longer Term

Respondents highlighted the need for integrated approaches to health provision by mainstreaming mental health throughout policies, action plans and strategies. In some countries, positive results had been achieved with political engagement, financial commitment and cross-sectoral coordination, but funding and resources remain a key challenge. They also stressed that support for mental health should go beyond outbreak situations to strengthen the entire mental health system, building on the progress made as part of emergency response to drive long-lasting and systemic changes that benefit mental health systems in the long term. For example, novel mental health services that emerged during the pandemic can help meet the mental health needs of the different population groups in a post-COVID-19 world. These new delivery modes enable broader access to previously excluded communities.

*“We are working on establishing a national tele-counselling and tele-psychiatric call centre at the national psychiatric referral hospital, … [This way] you don’t have to travel all the way to [the capital]”*.

[MoH MH focal point]

##### New Partnerships and Ways of Working

Some interviewees described how the COVID-19 emergency response had improved cross-sectoral coordination and collaboration and increased the representation of mental health across sectors. Some participants described how they had used stakeholder mapping to identify different stakeholders and how to engage them. Stakeholders included national government agents from different ministries, non-governmental organisations, international organisations, and UN agencies. Whilst some coordination groups were cross-sectoral multi-stakeholder, others were government-specific. In some cases, participants also mentioned that the private sector had supported the development of emergency responses.

*“We startedmapping now the mental health stakeholders, because we wanted to know what everyone is doing you know, UNHCR primarily based in the refugee camps and in with displaced populations, UNICEF is mainly children so we also involve the department of children services so that they can work together with UNICEF and the Ministry of Education, try to get people to like have different target groups because the pie is big, we’re trying to split it up and coordinate it better”*.

[MoH MH focal point]

Participants reported how regular meetings enabled a better mutual understanding of the scope and types of efforts made by the different stakeholders, improved resource distribution, and led to a more efficient and coordinated response. The involvement of political actors was seen as an enabler for multi-stakeholder coordination and the set-up of coordination bodies.

Participants also stressed the importance of the sustainability of multi-stakeholder coordination beyond emergencies. They noted how a return to more siloed and sectoral-specific coordination often impeded the integrated approach.

*“Our interaction during COVID, it was brilliant way of getting community engagement. … Our strategy over the years had been work with community actors so it becomes sustainable. They might even provide basic awareness raising, PFA in their local dialects”*.

[Civil society representative]

Working with the public was reported as a facilitator to addressing the stigma that might affect people suffering from mental health issues. Participants discussed how in the case of the COVID-19 outbreak, many people who tested positive for the disease had suffered stigma within their families and been excluded from their neighbourhoods or communities. Therefore, Communities were described as key actors in crisis response and awareness-raising.

The global pandemic spurred some governments to work with new actors in the crisis response, such as people with lived experience, which in turn may have had a positive effect on the way services are provided.

##### Drawing from Lessons Learned in Previous Outbreaks or Crises

Although it was noted previously that lessons had not systematically been learned from previous outbreaks, some participants did report that previous epidemics (notably Ebola outbreaks) had considerably reinforced the COVID-19 response. The different guidelines developed, referral pathways drawn out, and the training of frontline workers in earlier crises were instrumental in the COVID-19 response.

*“We canalso really give credit to the Ebola learning. So, lessons learned from Ebola set the pace for that, for the development of all those instruments, the referral pathway, documents developed. Everything we learn from Ebola, and integrated mental health”*.

[WHO MH focal point]

##### Sharing Experiences and Learning from Other Contexts

Almost all interviewees expressed a desire for better regional collaboration, drawing from experiences, and sharing lessons learned. This can help forge the way forward for countries contemplating how to address barriers to MHPSS action, both in the context of a crisis and in the longer term.

*“Sharingexperiences from other countries is so important. For example, countries presenting what they have done, so that one country can draw on the experience of another country”*.

[MoH MH focal point]

Participants particularly emphasised the importance of peer support, not only in terms of sharing technical skills, but also in fostering personal and professional motivation, which could easily be compromised when working in an under-resourced setting. Some participants outlined innovative solutions that could overcome logistical challenges, such as developing virtual peer-support networks and communities of practice.

*“Something can* *be developed either through tele-mentoring, a connection with other low and middle-income countries, particularly, you know, Africa region to share experiences with other professional policymakers, or clinicians to provide that kind of mentorship. (…) This could be a regional program allowing clinicians having issues in the facility to hook up to this system and get expert advice…”*

[Civil society representative]

Box 3 summarises the enablers and opportunities to improve the MHPSS component of crisis response.

Box 3 **Opportunities to improve the MHPSS component of crisis response.**

- Capitalising on the increased attention to mental health during COVID-19
- Promoting MHPSS integration in emergency response
- Better public understanding of mental health following the pandemic
- Integration of mental health in routine services and strengthening mental health systems in the longer term
- Sustaining multi-stakeholder coordination of MHPSS activities beyond emergencies
- Engaging communities and people with lived experience is a key to improving the MHPSS services provided and addressing the stigma.
- Drawing from lessons learned in previous crises to inform the preparedness and response to future public health emergencies
- Building regional networks to facilitate sharing experiences and learning between countries in the region.

## 4. Discussion

This mixed-methods study, using normative international standards for MHPSS implementation in outbreaks, has identified several common gaps in the COVID-19 response across African countries. Despite advances over recent years in the attention to mental health in global health policy and many national health systems, mental health was not prioritised in the response to COVID-19, in relation to both human and financial resourcing. This was despite learning on the importance of MHPSS from previous large-scale outbreaks such as the Ebola Virus Disease in West Africa [9]. Even when MHPSS was included in COVID-19 response strategies, only a minority of countries actually implemented these activities, and fewer countries monitored or evaluated them. Our research showed that the degree of implementation of MHPSS activities was less than 40% in more than half of the countries.

The lack of funding was consistently reported as a central challenge for MHPSS Leads who took part in this research. Similar results were reported by the WHO AFRO where only 25% of the respondent countries had ensured that full additional funding is available for MHPSS activities [10]. The lack of funding for MHPSS activities was also prominent in other WHO regions during the COVID-19 response [11]. Underinvestment in mental health is a chronic issue that goes beyond emergencies, and it includes both government and donor funding. In 2020, the government expenditure on mental health was 2.1% of the global median government expenditure on health [5]. Likewise, the development assistance for mental health represented only 0.4% of the total development assistance for health in 2015 [12]. Our research revealed some factors that might contribute to mental health underfunding. These factors include (1) the lack of political commitment and engagement, (2) low prioritisation of mental health and competing priorities in emergency situations, and (3) the lack of monitoring and evaluation mechanisms to measure the impact and effectiveness of MHPSS activities, which makes it hard to prove to funders that mental health is worth investing. In their report, Mackenzi and Kenser (2016) reported similar barriers to mental health funding such as competing priorities, concerns related to metrics, and the lack of knowledge about mental health [13].

Despite these challenges, new opportunities have emerged from the pandemic. Many respondents emphasised that the awareness of mental health had grown substantially. In some countries, this has become a new ministerial priority. New opportunities for partnership working across sectors in the pursuit of a coordinated MHPSS response were also created. Many respondents expected this to continue beyond the pandemic, though it remains to be seen if this renewed focus will lead to the strengthening of mental health systems and incorporation of MHPSS in future emergency preparedness, planning and response.

The pandemic has deepened existing inequities in access to mental health care. Resources were prioritised to other aspects of crisis response such as intensive care units and COVID-19 patient wards [14]. The difficulty in planning and implementing MHPSS support where mental health systems are already fragile or underfunded highlights the importance of strong pre-existing longer-term system structures. In this context, the importance of a “Build Back Better” approach is clear. The post-pandemic period is an opportunity to strengthen mental health systems as part of preparations for future public health emergencies [15]. Kola and colleagues [16] identified opportunities presented by the COVID-19 pandemic to reimagine global mental health and build more accessible services that contribute to Universal Health Coverage, shift towards non-coercive psychosocial interventions, and make use of new technologies to better meet the needs of neglected populations. This is particularly true for mental health and psychosocial support services.

This research has provided an overview of challenges and enablers in their broadest sense, across a diversity of contexts on the African continent. As such, we have prioritised breadth over depth. We found good coherence between the quantitative and qualitative aspects of our study. While the survey revealed generally low implementation levels of MHPSS activities, interviews with key stakeholders explained some of the key factors that impeded the implementation of MHPSS activities during the COVD-19 response. Further research could focus on particular innovations or challenges, or explore issues at a sub-regional level.

### 4.1. Recommendations

We suggest a number of recommendations based on the results of this study. Firstly, African public health institutions can harness lessons learnt, and include these in regional capacity building of mental health leaders to prepare for future emergencies. Secondly, increasing country preparedness by incorporating MHPSS in emergency planning, policy, training and integration could ensure that MHPSS retains a central role in future outbreak response. It is also imperative to establish an MHPSS pillar as part of the overall response, whose functions could be guided by the IASC recommendations. Thirdly, any resources allocated to MHPSS activities during the pandemic need to be sustained and built on—this includes funding for reform towards decentralised, task-sharing approaches and better integration across sectors. As our study revealed, it is vital to include monitoring and evaluation mechanisms to measure the effectiveness of MHPSS activities and prove that MHPSS is worth investing in. Now is the time to secure political commitment for investment in mental health through advocacy at national and international levels. All stakeholders involved in MHPSS should take advantage of the current momentum engendered by the COVID-19 crisis to increase priority to MHPSS during epidemics and other public health emergencies. Box 4 summarises the key recommendations to improve the MHPSS components in crisis response.

Box 4 **Recommendations to improve the MHPSS response in emergencies.**

- Establish an MHPSS response pillar as part of future responses to emergencies (guided by the IASC recommendations),
- Ensure that MHPSS components of national emergency preparedness and response plans include:
  - A feasible monitoring and evaluation framework
  - specific support for children and families
  - regular community engagement during the response
  - allocated resources to implement MHPSS components
- Stress test (through exercises and desktop scenarios) the MHPSS components of the national emergency response plans (particularly testing the capacity of human and financial resources) and refine plans accordingly
- Sensitize national leaders to the importance of MHPSS in emergency preparedness and response and lessons learned from COVID-19
- Undertake an in-depth review of MHPSS components of the national response to COVID-19 and identify lessons learnt
- Improve data and information systems in routine national mental health systems to improve this function during emergencies.

### 4.2. Limitations

There are several limitations of this study. While the study did use international normative guidance as assessment criteria, surveys were based on self-reported data from the perspective of national mental health leads. As such, it is a guide to where people leading mental health components of outbreak response feel gaps lie, rather than an objective measure. Respondents were, however, extremely well placed to have this knowledge, and were informed that results would be anonymised, and this should mitigate bias. Attempting to validate results by triangulating between stakeholders in the same country may have given insights into other forms of self-assessment bias. Despite our best attempts, we were not able to obtain information from all countries on all variables across the continent. Response to the survey may have depended on the strength of the national MHPSS response to COVID-19 and so could also bias our findings. Responses may overlook potential significant variability within countries (e.g., regional differences, with urban contexts probably better reported).

## 5. Conclusions

A limited number of recommended MHPSS activities during the COVID-19 pandemic were planned in countries across Africa, with an even smaller proportion being actually implemented. The implementation level of MHPSS activities was below 50% in most countries. Given the risk of emergencies being high in many African countries, this represents a serious failure in global public health. The most implemented MHPSS activities were establishing coordination groups (57%) and developing MHPSS strategy (45%). This represents some progress in recent years, but implementation and monitoring and evaluation were much weaker. We recognised several factors that limit the integration of MHPSS into emergency response. These factors include the lack of political commitment and low prioritisation of mental health during emergencies, as it was seen as a “less important” issue during the COVID-19 pandemic, where more importance was given to infection prevention and control (IPC). However, there are signs of optimism, as mental health has gained some attention during COVID-19. It is critical to build on this to integrate mental health into emergency preparedness and response and strengthen mental health systems in the long term in the post-pandemic world.

## Figures and Tables

**Table 1:** Colour coding of Likert scale responses.

Survey Response
Fully implemented
Almost fully implemented
Somewhat implemented
Not at all implemented
Do not know
Blank response

## Data Availability

The data presented in this study are available on request from the corresponding author.

## Supplementary Materials

The following supporting information can be downloaded at: www.mdpi.com/1660-4601/19/15/9313/s1, File S1: the full survey used in the research; File S2: Interviews topic guide.
