# Supplementary material for: Challenges and Opportunities for Mental Health and Psychosocial Support in the COVID-19 Response in Africa: A Mixed-Methods Study
Source: Int J Environ Res Public Health. 2022 Jul 29;19(15):9313. doi: 10.3390/ijerph19159313 (PMC9368294; doi:10.3390/ijerph19159313)
Supplement: Supplementary file 1 [file ijerph-19-09313-s001.zip › Supplementary File S1.pdf]

Mental Health and Psychosocial Support response for COVID-19 in Africa

0%

0% complete

Page 1: Introduction

Thank you for your participation in this short survey.

Completion time is around 20 minutes. Your responses will inform work by WHO AFRO, WHO EMRO, Africa CDC, West African Health Organisation (WAHO) and London School of Hygiene and Tropical Medicine (LSHTM) to understand challenges in addressing Mental Health and Psychosocial Support (MHPSS) needs as part of the COVID-19 response in Africa.

This survey is being sent to 2-3 key stakeholders per country. It is part of a broader programme of work: "Strengthening Public Mental Health in Africa in response to COVID-19".

Results of this survey will be used to design a programme for strengthening capacity and increasing resources for MHPSS as part of outbreak response in African countries. Survey participants will be invited to participate in the training and networking opportunities which are part of this project.

Survey participants are invited to provide their email for the purpose of follow-up research and training opportunities. These will be stored separately from survey responses, which will be anonymous. If you have any queries about this survey please contact Alice Walker: Alice.Walker2@lshtm.ac.uk

If you have queries about Mental Health and Psychosocial Support in response to COVID-19, please contact

Florence Baingana (WHO AFRO): bainganaf@who.int

Mohammed Abdulaziz (Africa CDC): MohammedA@africa-union.org

Page 2: Background information

1.1.What is your email address?

2.2.Which country do you work in? Required

a.2.a.Which institution do you work for? Required

- National Government department
- National Public Health Institute
- WHO
- WAHO
- Africa CDC
- Other

i.2.a.i.If you selected Other, please specify:

ii.2.a.ii.Which Government department?

- Ministry of Health
- Ministry of Social/Family Affairs
- Ministry of Education
- Ministry of Finances
- Ministry of Foreign Affairs
- Other

a.2.a.ii.a.If you selected Other, please specify:

iii.2.a.iii.What is your role?

iv.2.a.iv.Are you a focal point for mental health in the COVID-19 response within your organisation?

- Yes
- No

3.3.Was the Mental Health and Psychosocial Support (MHPSS) response to COVID-19 in your country informed by international or regional guidance?

- Yes
- Partially
- No
- Don't know

a.3.a.If so, please specify which guidance was used

b.3.b.If not, what were the reasons?

- No guidance available
- Guidance not relevant
- Lack of capacity or resources to implement guidance
- Other

i.3.b.i.If you selected Other, please specify:

Page 3: Instructions

The following questions ask you to rate the degree of implementation of recommended MHPSS activities as part of the COVID-19 response, selecting from the following options:

-Fully implemented

-Almost fully implemented *(implemented with some gaps)*
-Somewhat implemented *(some measures taken but significant gaps)*

-Not at all implemented

-Do not know

Responses are anonymous, and results will be used to design a programme for strengthening capacity and increasing resources to support MHPSS as part of the COVID-19 response

Page 4: Assessing Need

4. To what extent has a rapid MHPSS needs assessment been performed as part of the COVID-19 response in your country? *Required*

- ○ Fully
- ○ Almost fully
- ○ Somewhat
- ○ Not at all
- ○ Do not know

a. If so, at which level was this conducted?

- ○ National level
- ○ Subnational level
- ○ Not Applicable

b. Which of the following were considered? Please tick all that apply

- ☐ Population needs
- ☐ Vulnerable groups,
- ☐ Resources
- ☐ Capacity gaps
- ☐ Training needs
- ☐ Other

ii. Please provide any other information you feel is relevant (optional)

Page 5: Response planning

5.5.**To what extent has an MHPSS coordination group, technical working group, or response unit been established?** Required

- Fully
- Almost fully
- Somewhat
- Not at all
- Do not know

a.5.a.Is this group integrated or standalone

- Integrated
- Standalone
- Other

i.5.a.i.If you selected Other, please specify:

ii.5.a.ii.Who is part of this group? Tick all that apply

- Ministry of Health
- Ministry of Social/Family Affairs
- Ministry of Education
- Ministry of Finances
- Ministry of Foreign Affairs
- United Nations Agencies
- Governmental entity responsible for substance use
- International non-governmental Organizations
- Non-governmental Organizations

- [ ] Service users' representatives
- [ ] Other

a.5.a.ii.a.Who within the MoH was part of this group?

b.5.a.ii.b.If you selected Other, please specify:

6.6.To what extent has an MHPSS strategy for COVID-19 been developed? Required

- ( ) Fully
- ( ) Almost fully
- ( ) Somewhat
- ( ) Not at all
- ( ) Do not know

a.6.a.Was this strategy developed at national or subnational level?

- ( ) National
- ( ) Subnational/District
- ( ) Both
- ( ) Do not know
- ( ) Other

i.6.a.i.If you selected Other, please specify:

b.6.b.To what extent has this strategy been implemented?

- [ ] Fully

- [ ] Almost fully
- [ ] Somewhat
- [ ] Not at all
- [ ] Do not know

c.6.c.Is additional funding available for strategy implementation?

- ( ) Yes
- ( ) No
- ( ) Don't know

d.6.d.Who is responsible for strategy development and implementation?

e.6.e.Please provide any further information you feel is relevant

7.7.To what extent has information from needs assessments and service analysis been used to ensure there is a system to identify and provide care to people with common and severe mental health and substance misuse conditions? This includes ensuring every health facility has at least one person trained in MHPSS

- ( ) Fully
- ( ) Almost fully
- ( ) Somewhat
- ( ) Not at all
- ( ) Do not know

Page 6: Part 3: Delivering Support

8.8.To what extent have MHPSS activities been integrated into other sectoral response activities? Required

- Fully
- Almost fully
- Somewhat
- Not at all
- Do not know

a.8.a.Which sectors?

- Education
- Social protection
- WASH
- Nutrition
- Gender
- Disability and Inclusion
- Other

i.8.a.i.If you selected Other, please specify:

9.9.To what extent are there functional referral pathways to MHPSS services from other sectors? (An example of one such pathway might include a referral pathway for those with mental health needs identified among recipients of social protection services) Required

- Fully
- Almost fully
- Somewhat
- Not at all

- [ ] Do not know

a.9.a.Were these developed as part of the COVID-19 response?

- [ ] Yes
- [ ] No
- [ ] Do not know

b.9.b.Please provide any further information you feel is relevant

10.10.To what extent are accurate information and communication materials on MHPSS available and accessible? *Required*

- [ ] Fully
- [ ] Almost fully
- [ ] Somewhat
- [ ] Not at all
- [ ] Do not know

a.10.a.Who co-ordinated this effort?

b.10.b.What materials were used? (Tick all that apply)

- [ ] Social media
- [ ] Posters
- [ ] Pamphlets
- [ ] Radio
- [ ] Television
- [ ] SMS

Other

i.10.b.i.If you selected Other, please specify:

11.11.To what extent have frontline workers (including nurses, ambulance drivers, volunteers, case identifiers, teachers, community leaders, and other non-health workers) been trained in principles of psychosocial care and psychological first aid? *Required*

Fully

Almost fully

Somewhat

Not at all

Do not know

a.11.a.What does this training include? Tick all that apply

Essential psychosocial care principles

Psychological first aid

Making referrals

Other

i.11.a.i.If you selected Other, please specify:

ii.11.a.ii.Who is co-ordinating and delivering this training?

12.12.To what extent do frontline workers responding to COVID-19 have access to psychological support? *Required*

Fully

- Almost fully
- Somewhat
- Not at all
- Do not know

a.12.a.Please describe support available

13.13.To what extent has specific support for children and families been established as part of the COVID-19 response? *Required*

- Fully
- Almost fully
- Somewhat
- Not at all
- Do not know

a.13.a.What kinds of support are available?

- Activity packs
- Learning materials
- Communication materials
- Child and Adolescent MHPSS services
- Child-friendly spaces
- Other

i.13.a.i.If you selected Other, please specify:

14.14.To what extent have community-based forms of mental health and psychosocial support been established? *Required*

- Fully
- Almost fully
- Somewhat
- Not at all
- Do not know

a.14.a.How is support provided?

- Social media
- Religious groups
- Educational institutions
- Community health workers
- Other

i.14.a.i.If you selected Other, please specify:

15.15.To what extent have MHPSS measures been established to reduce the negative impact of social isolation? *Required*

- Fully
- Almost fully
- Somewhat
- Not at all
- Do not know

a.15.a.For which groups?

- Cases and contacts in quarantine sites
- Medically vulnerable groups (eg elderly, those with comorbidities)
- Carers

General population

b.15.b.Please describe these measures

16.16.To what extent have COVID-19 and MHPSS protocols been developed for funerals and mourning? *Required*

Fully

Almost fully

Somewhat

Not at all

Do not know

Page 7: Part 4: Monitoring and Evaluation

17. To what extent have monitoring and evaluation mechanisms to measure MHPSS activities been established? *Required*

- Fully
- Almost fully
- Somewhat
- Not at all
- Do not know

a. Please provide any further information: Who leads this process, which indicators are measured?

b. Will an evaluation of the MHPSS aspects of the response be performed?

- Yes
- No
- Do not know

Page 8: Part 5: Learning and review

18. What were the key successes or enablers to the MHPSS aspects of the response? *Required*

19. What were the key challenges or bottlenecks in planning and delivering MHPSS activities? *Required*

20. Did response co-ordinators have access to any of the following training or support when planning the MHPSS response? Tick all that apply *Required*

- [ ] Webinars (WHO, WAHO, CDC)
- [ ] International guidance
- [ ] Online tools or resources
- [ ] Face-to-face training
- [ ] No training or support available
- [ ] Other

21. What additional support would be useful for response coordinators to strengthen the MHPSS response? *Required*
