# Supplementary material for: Challenges and Opportunities for Mental Health and Psychosocial Support in the COVID-19 Response in Africa: A Mixed-Methods Study
Source: Int J Environ Res Public Health. 2022 Jul 29;19(15):9313. doi: 10.3390/ijerph19159313 (PMC9368294; doi:10.3390/ijerph19159313)
Supplement: Supplementary file 1 [file ijerph-19-09313-s001.zip › Supplementary File S2.pdf]

Supplementary information S2 : Interview topic guide

Theme	Stem questions	Potential follow up questions:
**Planning and coordination**	What is your role?	
	Can you tell me about how the MHPSS aspects of COVID-19 outbreak response were coordinated?	
	Was there previous experience of outbreak response at the national or subnational level in your country?	How did MHPSS aspects of the response to COVID-19 compare to previous outbreaks?<br><br>What lessons were learned in terms of integrating MHPSS in outbreak responses?<br><br>Did MHPSS work as part of previous outbreak responses help with the response this time? If so, how?
**Delivering support**	What were the main MHPSS needs in response to COVID-19?	How were these assessed?<br><br>Were they different to previous outbreaks or emergencies?
	What support has been provided	
	What needs have not been addressed?<br><br>If not mentioned, ask about HCWs, children, carers	Why/why not?<br>What needs to be done to address these?
	Was MHPSS integrated into other sectoral response (eg social protection, education etc)	How did this happen?<br>How/why not?<br>What would have helped?
	Has any support been planned to address the longer term impacts of COVID-19 on mental health?	
	Will MHPSS efforts in response to COVID-19 strengthen public mental health after the outbreak?	If yes – how?<br>If no – why not?

**Enablers**	Can you tell us more about what you felt went well in the mental health response overall?	Why was that the case?<br>What could have helped you more?<br>What should be done differently?
	What were the factors that supported these achievements?	How did these help?<br>Were these easy/difficult to get?
	What could be done to build on these successes?	
**Barriers**	What were the overall challenges faced in coordinating the MHPSS response?	What was/is needed to address these challenges?
	[if needed] What were the factors that contributed to these challenges?	
**Specific factors (if not covered above)**	In terms of outbreak preparedness, was MHPSS addressed in preparedness planning?	
	Was there political commitment to address MHPSS as part of the response?	If no - why not?<br>If yes – how was this achieved?<br><br>What would be most useful to support political advocacy in your country?
	Was funding of MHPSS appropriate for the level of need, relative to other needs	If yes – how was funding secured<br>If no – why not?<br><br>What would be needed to make the case for extra funding?
	What are your country's human resource needs to be able to better respond to MHPSS needs in outbreaks?	
**Existing support**	Was national or international guidance used to help plan the response?	Was this national or international?<br><br>Were these guidelines useful in your context? Why/why not?<br><br>What were the barriers in guidance implementation?

	Which other actors supported the mental health aspects of the response?<br><br>If not mentioned ask about: multilateral agencies, civil society, the media	What support was provided?<br><br>Was it helpful? Why?<br><br>What more was needed?
**Support needs**	What training have you found most useful for your role?	Do you have any further support needs?
	What is needed to support implementation of MHPSS support as part of outbreak responses?	If not mentioned ask about:<br><br>Implementation guidance – eg Minimum service packages<br><br>Support with strategy development<br><br>Sharing best practice
